# Lactose intolerance among severely malnourished children with diarrhoea admitted to the nutrition unit, Mulago hospital, Uganda

**DOI:** 10.1186/1471-2431-10-31

**Published:** 2010-05-06

**Authors:** Richard Nyeko, Israel Kalyesubula, Edison Mworozi, Hanifa Bachou

**Affiliations:** 1 Department of Paediatrics and Child Health, St. Mary's hospital Lacor, Gulu, Uganda P.O Box 180, Gulu, Uganda; 2 Department of Paediatrics and Child Health, College of Health Sciences, Makerere University, Kampala, Uganda, P.O Box 7072, Kampala, Uganda

## Abstract

**Background:**

Lactose intolerance is a common complication of diarrhoea in infants with malnutrition and a cause of treatment failure. A combination of nutritional injury and infectious insults in severe protein energy malnutrition reduces the capacity of the intestinal mucosa to produce lactase enzyme necessary for the digestion of lactose.

The standard management of severe malnutrition involves nutritional rehabilitation with lactose-based high energy formula milk. However, some of these children may be lactose intolerant, possibly contributing to the high rate of unfavorable treatment outcomes. This study was therefore designed to establish the prevalence of lactose intolerance and associated factors in this population.

**Methods:**

A descriptive cross sectional study involving 196 severely malnourished children with diarrhoea aged 3-60 months was done in Mwanamugimu Nutrition Unit (MNU), Mulago hospital between October 2006 and February 2007.

**Results:**

During the study period, 196 severely malnourished children with diarrhoea were recruited, 50 (25.5%) of whom had evidence of lactose intolerance (stool reducing substance ≥ 1 + [0.5%] and stool pH < 5.5) and it occurred more commonly in children with kwashiorkor 27/75 (36.0%) than marasmic-kwashiorkor 6/25 (24.0%) and marasmus 17/96 (17.7%). Oedematous malnutrition (p = 0.032), perianal skin erosion (p = 0.044), high mean stool frequency (p = < 0.001) and having ≥2 diarrhoea episodes in the previous 3 months (p = 0.007) were the independent predictors of lactose intolerance.

Other factors that were significantly associated with lactose intolerance on bi-variate analysis included: young age of 3-12 months; lack of up to-date immunization; persistent diarrhoea; vomiting; dehydration, and abdominal distension. Exclusive breastfeeding for less than 4 months and worsening of diarrhoea on initiation of therapeutic milk were the other factors.

**Conclusions:**

The prevalence of lactose intolerance in this study setting of 25.5% is relatively high. Routine screening by stool pH and reducing substances should be performed especially in the severely malnourished children with diarrhoea presenting with oedematous malnutrition, perianal skin erosion, higher mean stool frequency and having had ≥2 diarrhoea episodes in the previous 3 months.

Use of lactose-free diets such as yoghurt should be considered for children found to have evidence of lactose intolerance and whose response on standard therapeutic milk formula is poor.

## Background

Lactose intolerance is a common complication of diarrhoea in infants with malnutrition [1], and a cause of treatment failure [2,3]. Prevalences of secondary lactose intolerance have been reported to range from 26% to as high as 100% of affected children in some patients in different settings [4-6]. Lactase activity is reduced in many patients with kwashiorkor even at early ages, and carbohydrate intolerance occurs more frequently in children with kwashiorkor (48.3%) than in those with marasmus (15%), marasmic-kwashiorkor (20%) and healthy controls (23.5%) [7]. Kwashiorkor is also associated with variable degrees of malabsorption that complicate the nutritional rehabilitation of the patients. As a consequence, patients with this form of malnutrition also have vitamin and mineral depletion of variable severity and this contributes to further damage to their small intestinal mucosa and to the abnormal proliferation of bacteria in their gut and to the appearance of immune deficiencies.

Children with severe protein-energy malnutrition commonly have a reduced activity of intestinal lactase, the enzyme responsible for the digestion of lactose [8,9], and it has been suggested that feeding this disaccharide can retard nutritional recovery [10]. Secondary lactase deficiency can present at any age but is more common in infancy [11,12]. This contrasts with the risks in normal children as demonstrated by Gabr and colleagues in Egypt: 12% in the age group 6 months to 2 years, 32% in the age group 2-5 years, 32% in the age group 5-9 years, and 80% in the age group 9-12 years [13].

The manifestations of lactose intolerance are watery acidic stools, abdominal distension and excessive flatus [14]. Perianal skin erosion is also observed frequently and is caused by contact of acidic, watery stools with the skin [15,16]. Lactose intolerance rates are significantly increased in children with a history of recent diarrhoea [17], and dehydration due to osmotic diarrhoea may be common [14].

In one study, milk intolerance presenting as diarrhoea was significantly more common in children with giardiasis [18]. Similarly, Pettoello and colleagues in Italy concluded that the occurrence of lactose malabsorption of nutritional relevance was common in children suffering or having suffered from giardiasis (45%) [19].

The mortality of severely malnourished children is very high (24% in the setting in which this study was carried out) [20]. Part of this is due to dehydration which is aggravated by lactase deficiency. Little is known concerning the magnitude of lactose intolerance in this study setting, yet the standard of care involves the use of lactose-based high energy formula milk. This study was therefore designed to establish the prevalence of lactose intolerance and associated factors in this population.

## Methods

### Study setting

The study was undertaken at Mwanamugimu Nutrition Unit (MNU) of Mulago hospital, Kampala, the national referral and teaching hospital for Makerere University College of Health Sciences. The department of Paediatrics and Child Health is one of the largest departments in the hospital, admitting over 10 000 children annually. The MNU is a 72-bed capacity unit specialized in managing children with severe acute malnutrition with an average monthly admission of 50-70 patients, about 50% of whom present with diarrhoea, and this varies according to seasons.

### Study design

This was a descriptive cross-sectional study. The sample size was calculated using the Leslie Kish formula [21] and a prevalence of 15% as found by Tolboom in marasmic Besotho children [7].

The study population consisted of severely malnourished children with diarrhoea aged 3-60 months admitted to MNU, Mulago hospital during the study period. The WHO classification of severe malnutrition was used: A severely malnourished child was one whose weight-for-height was less than -3SD or less than 70% of the median National Centre for Health Statistics (NCHS)/WHO reference median (severe wasting), or who had bilateral pitting pedal oedema. The Wellcome classification was used to classify a malnourished child as having kwashiorkor (defined as weight-for-age between 60-80% of the median in the presence of bilateral pitting pedal edema), marasmus (weight-for-age < 60% in the absence of edema), or marasmic-kwashiorkor (weight-for-age < 60% in the presence of bilateral pitting pedal edema). Children who were already on modified lactose-free diet were excluded.

### Data collection/study procedure

The authors of this study explored the incidence of lactose intolerance in the study population on the basis of a pre-coded and pre-tested structured questionnaire and tests in fresh stools of the participants at least 24 hours after initiating standard lactose-based therapeutic milk diet, with measurements of stool pH and presence of reducing substances in faeces. The test was considered positive if stool reducing substances were equal to or more than 1 + (= 0.5%) and stool pH less than 5.5. Stool microscopy was performed for fat globules, ova/cysts, parasites (giardiasis), pus cells and yeasts (Candida albicans). Recruitment into the study was by consecutive enrollment of children who fulfilled the inclusion criteria. A quick assessment for complications including hypothermia, dehydration and/or shock and severe infections was done by the principal investigator. Any necessary resuscitation management was instituted before recruitment into the study. The purpose of the study was explained to the parents/caretakers, including pretest HIV counseling and informed consent were obtained for participation in the study. Children whose caretakers declined HIV test were still recruited if they were willing to participate and consented for the study.

### Laboratory procedure

Blood samples were obtained for an HIV test. Post test counseling was done as soon as results were obtained. All children found to be HIV positive were referred to the Paediatric Infectious Disease Clinic (PIDC) for appropriate management and their mothers referred to the adult clinic for appropriate care as well. The HIV test was done under an arrangement of routine counseling and testing (RCT) that was already operational in the unit. CD4 counts were not done.

Stool pH was tested using narrow range pH papers after dissolution of the fresh stool specimen in distilled water, while presence of reducing substance was tested by use of Benedict's solution.

Quality control was ensured by using one qualified laboratory technologist to carry out the analysis of samples using an established reputable laboratory of Mulago National Referral Hospital.

### Data management and analysis

Data was coded and entered into a computerized database using Epidata 3.1. All data collected was cross-checked for completeness and accuracy and cleaned before analysis. The analysis was done using the Statistical Package for Social Sciences (SSPS II) software. Categorical variables were summarized as frequencies and proportions, while continuous variables as means, median and standard deviations (SD).

In the bi-variate analysis, odds ratios, 95% confidence interval (CI), and chi-square test were used to measure the strength of association between the factors considered and the dependent variable, while the student's t-test was used for continuous variables.

Multivariate analysis using logistic regression was used to determine the factors that were independently associated with lactose intolerance. P-value < 0.05 was considered for statistical significance. Results were summarized in texts, tables and bar graphs.

### Ethical considerations

The study was approved by the Ethics and Research Committee of the School of Health Sciences, Makerere University, and the Uganda National Council for Science and Technology. Voluntary informed consent was obtained from the parents/caretakers before participating in the study. Confidentiality was observed throughout the study. All children found to be HIV positive were referred to PIDC and their mothers to the adult HIV clinic for further management.

## Results

The study included 196 children 3-60 months of age (mean age 15.5 months and median 12 months). Sixty four (32.7%) of the study children were HIV positive and only one of them was on antiretroviral therapy, 113 (57.7%) were males and only 50.0% (98/196) had up to-date immunization.

**Figure 1:**
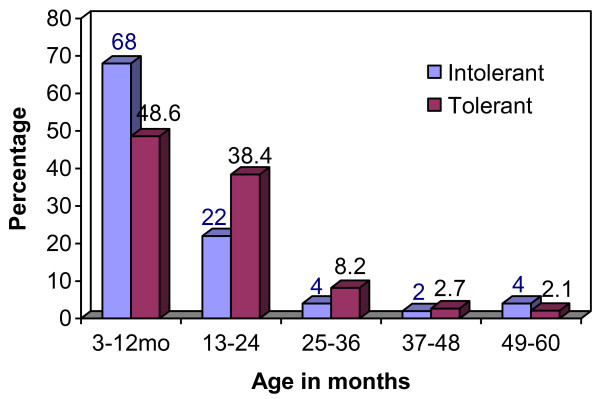
**Age distribution by lactose tolerance**.

The prevalence of lactose intolerance was 25.5% by these criteria and was highest among the children in the age group 3-12 months (68.0%), with 90% of the cases occurring in the first two years of life. The prevalence then markedly reduced after 24 months of age, a trend that mirrors the prevalence of severe malnutrition (Figure 1).

Lactose intolerance occured more commonly in children with kwashiorkor {27/75 (36.0%), [p=0.011]} than marasmic-kwashiorkor {6/25 (24.0%), [p=1.000]} and marasmus {17/196 (17.7%), [p=0.021]} (Table 1).

**Table 1:** Lactose tolerance by type of malnutrition

Characteristics	Lactose intolerant N = 50(%)	Lactose tolerant N = 146(%)	OR	95% CI	p-value
Kwashiorkor:					
Yes	27(36.0)	48(64.0)	2.40	1.25-4.61	0.011*
No	23(19.0)	98(81.0)			
#Marasmic-kwash:					
Yes	6(24.0)	19(76.0)	0.91	0.34-2.43	1.000
No	44(25.7)	127(74.3)			
Marasmus:					
Yes	17(17.7)	79(82.3)	0.44	0.22-0.85	0.021*
No	33(33.0)	67(67.0)			

*P-value significant (< 0.05), OR = Odd's ratio, CI = 95% confidence interval,

# Marasmic-kwashiorkor

At bivariate analysis, child's age and immunization status (Table 2), duration of exclusive breastfeeding and effect of starting therapeutic milk (Table 3) were all significantly associated with lactose intolerance. Also significant were duration of diarrhoea, diarrhoea episodes in the previous 3 months and stool frequency (Table 4). Vomiting, body temperature, hydration status, presence of oedema, perianal skin erosion, and abdominal distension were the other significant clinical characteristics (Table 5).

**Table 2:** Baseline characteristics of the study population and lactose intolerance

Characteristics	Lactose intolerant N = 50(%)	Lactose tolerant N = 146(%)	OR	95% CI	p-value
**Sex**:					
Male	27(23.9)	86(76.1)	0.82	0.43-1.56	0.545
Female	23(27.7)	60(72.3)			
**Age in months**:					
3-12	34(32.4)	71(67.6)	2.25	1.14-4.42	0.018*
Above 12	16(17.6)	75(82.4)			
**Birth order**:					
First	18(31.0)	40(69.0)	1.49	0.75-2.95	0.250
≥Second	32(23.2)	106(76.8)			
**Immunization status**:					
Not up to-date	39(39.8)	59(60.2)	5.23	2.48-11.03	< 0.001*
Up to-date	11(11.2)	87(88.8)			
**ΔMeasles in the previous 3 months**:					
Yes	18(35.3)	33(64.7)	1.90	0.94-3.83	0.070
No	31(22.3)	108(77.7)			
**¶HIV status**:					
HIV+ve	19(29.7)	45(70.3)	1.42	0.72-2.80	0.315
HIV-ve	28(23.0)	94(77.0)			

*P-value significant (< 0.05), OR = Odd's ratio, CI = 95% confidence interval

**Δ **Some caretakers were unsure, hence the ones considered (190/196) do not add up to 196

**¶ **Only those with confirmed HIV status were considered (186/196) hence they do not add up to 196. HIV by rapid test for age ≥18 months and DNA PCR for age < 18 months.

**Table 3:** Feeding practices associated with lactose intolerance

Characteristics	Lactose intolerant N = 50(%)	Lactose tolerant N = 146(%)	OR	95% CI	p-value
**Δ Still breastfeeding**:					
Yes	11(24.4)	34(75.6)	0.87	0.40-1.90	0.719
No	34(27.2)	91(72.8)			
**#Duration of EBF:**					
< 4 months	25(35.7)	45(64.3)	2.24	1.16-4.33	0.015*
≥4 months	25(19.8)	101(80.2)			
**Ever had problems with cow's milk:**					
Yes	3(50.0)	3(50.0)	3.04	0.59-15.59	0.175Ψ
No	47(24.7)	143(75.3)			
**¶Effect of starting therapeutic milk:**					
Diarrhea worsened	25(35.2)	46(64.8)	2.88	1.41-5.86	0.003*
No effect	17(15.9)	90(84.1)			
**Therapeutic milk:**					
F75	41(25.6)	119(74.4)	1.03	0.45-2.38	0.938
F100	9(25.0)	27(75.0)			

*P-value significant (< 0.05), OR = Odd's ratio, CI = 95% confidence interval

**Δ **Only children in the breastfeeding age range (3-24 months) were considered; hence they do not add up to 196.

**# **Duration of exclusive breastfeeding, Ψ Fisher's Exact Test

**¶ **Only for those who had diarrhoea at admission, hence they do not add up to 196, (178/196).

**Table 4:** Diarrhoea characteristics associated with lactose intolerance

Characteristics	Lactose intolerant N = 50(%)	Lactose tolerant N = 146(%)	OR	95% CI	p-value
**Duration:**					
≥14 days	26(34.2)	50(65.8)	2.08	1.08-3.99	0.026*
< 14 days	24(20.0)	96(80.0)			
**Watery stool:**					
Yes	34(26.8)	93(73.2)	1.21	0.61-2.40	0.583
No	16(23.2)	53(76.8)			
**Blood in stool:**					
Yes	2(40.0)	3(60.0)	1.99	0.32-12.24	0.603Ψ
No	48(25.1)	143(74.9)			
**Antibiotic use during diarrhoea:**					
Yes	25(24.8)	76(75.2)	0.92	0.48-1.75	0.802
No	25(26.3)	70(73.7)			
**Use of local herbs:**					
Yes	13(26.0)	37(74.0)	1.040	0.50-2.16	0.927
No	37(25.3)	109(74.7)			
**Diarrhea episodes in the previous 3 mo**					
≥2	40(47.1)	45(52.9)	8.98	4.13-19.52	< 0.001*
One	10(9.0)	101(91.0)			
**Fat globules in stool**					
Yes	3(60.0)	2(40.0)	4.60	0.75-28.34	0.106Ψ
No	47(24.6)	144(75.4)			
**Yeasts in stool (Candida albicans):**					
Yes	29(27.6)	76(72.4)	1.27	0.67-2.43	0.467
No	21(23.1)	70(76.9)			
**Pus cells in stool:**					
Yes	6(20.0)	24(80.0)	0.693	0.27-1.81	0.452
No	44(26.5)	122(73.5)			
**¶Frequency of stool in 24 hrs:**	9.48(2.35)	5.81(2.20)			< 0.001*

*P-value significant (< 0.05), OR = Odd's ratio, CI = 95% confidence interval,

**¶ **Student t-test used for mean stool frequencies (standard deviations). Ψ Fisher's Exact Test

**Table 5:** Clinical characteristics of the severely malnourished children

Characteristics	Lactose intolerant N = 50(%)	Lactose tolerant N = 146(%)	OR	95% CI	p-value
History of fever	31(62.))	92(63.0)	0.96	0.49-1.86	0.898
History of vomiting	35(70.0)	76(52.1)	2.15	1.08-4.27	0.027*
History of cough	35(70.0)	116(79.5)	0.60	0.29-1.25	0.170
Temperature (≥37.5°C)	10(20.0)	41(28.1)	0.64	0.29-1.40	0.261
Temperature (≤35°C)	12(24.0)	10(6.8)	4.30	1.72-10.70	0.001*
Oedema	33(66.0)	67(45.9)	2.29	1.17-4.47	0.014*
Severe pallor	1(2.0)	3(2.1)	0.97	0.10-2.40	1.000Ψ
Dehydration	31(62.0)	60(41.1)	2.34	1.21-4.52	0.011*
Oral thrush	11(22.0)	38(26.0)	0.80	0.37-1.72	0.570
Lymphadenopathy	4(8.0)	11(7.5)	1.07	0.32-3.52	1.000Ψ
Perianal erosion	35(70.0)	28(19.2)	9.83	4.73-20.44	< 0.001*
Abdominal distension	20(40.0)	24(16.4)	3.39	1.66-6.93	0.001*
Hepatomegally	21(42.0)	47(32.2)	1.53	0.79-2.95	0.209
Splenomegally	4(8.0)	8(5.5)	1.50	0.43-5.21	0.506Ψ

*P-value significant(< 0.05), OR = Odd's ratio, CI = 95% confidence interval, Ψ Fisher's exact test

At multivariate analysis, oedematous malnutrition, perianal skin erosion, high mean stool frequency and having two or more diarrhoea episodes in the previous 3 months were the independent predictors of lactose intolerance (Table 6).

**Table 6:** Logistic regression model for factors independently predicting lactose intolerance

Characteristics	Odd Ratio	95% CI	p-value
Immunization status	2.60	0.85-7.93	0.093
Diarrhea episodes in previous 3 months	4.88	1.53-15.55	0.007*
Oedema	3.40	1.11-10.40	0.032*
Perianal erosion	3.07	1.03-9.16	0.044*
Frequency of stool/24 hrs	0.59	0.48-0.74	< 0.001*

* P-value significant (< 0.05), OR = Odd's ratio, CI = 95% confidence interval

## Discussion

The 25.5% prevalence of lactose intolerance in the 196 severely malnourished children with diarrhoea accords with a previous finding in a Senegalese study of 26% [4], but lower than that reported in other studies [5,6], a difference that might be explained by the difference in sample size and study population. Nonetheless, the prevalence of lactose intolerance in the current study is still significantly high, a fact that could be a plausible contributing factor to the unfavorable outcome that has remained a challenge in the management of severely malnourished children, more so those with diarrhoea.

Evidence of lactose intolerance occurred more frequently in children with kwashiorkor 27/75 (36.0%) than in those with marasmic-kwashiorkor 6/25 (24.0%) and marasmus 17/96 (17.7%), a finding consistent with that by Tolboom and colleagues [7]. This might not be surprising and seems to reinforce the fact that kwashiorkor is associated with variable degrees of malabsorption, with protein and energy as well as vitamin and mineral deficiencies of variable severity that contributes to intestinal mucosal damage, in addition to oxidative stress. Failure of the intestinal barrier from bacterial overgrowth and passage of lipopolysaccharide to the systemic circulation and uncontrolled stimulation of the inflammatory mechanisms probably also have a part to play.

Most children (68%) with lactose intolerance were infants 3-12 months, a finding consistent with several other studies [3,12]. This, however, contrasts with the risks in normal children as demonstrated by Gabr and colleagues in Egypt [13], a difference attributed to the fact that in normal children, lactose intolerance is mainly due to primary lactase deficiency which increases with age (becomes apparent by 5 years of age), as opposed to secondary lactase deficiency which, though can present at any age, is more common in infancy [11]. In our study, there was a gradual decrease in the prevalence of lactose intolerance from 68% in the age group 3-12 to 2% in the 37-48 age groups, with a slight rise thereafter to 4% among the 49-60 age groups which could be attributed to underlying primary lactose intolerance which becomes apparent by 5 years of age, possibly aggravated by the diarrhoea and severe malnutrition.

Children with lactose intolerance were more likely to have a higher mean stool frequency (≥8 motions in 24 hour period) [p < 0.001], a finding consistent with that by Ozmert and colleagues in Turkey [12]. This is not surprising since unabsorbed lactose that is not metabolized by the colonic bacteria to organic acids would remain in the colonic lumen and lead to osmotic diarrhoea. Furthermore, undigested lactose may attract such an amount of water in the jejunum-ileum that the colon cannot handle, with the speed of transit also a contributing factor in the whole process.

High prevalence of lactose intolerance among children having had two or more diarrhoea episodes in the previous 3 months as found in this study has also been reported elsewhere [17]. Recurrent episodes of diarrhoea result in repeated disruption of the intestinal villi with shortened regeneration and maturation time, predisposing to intestinal lactase deficiency.

Lactose intolerance was more likely in children with persistent diarrhoea (34.2%) compared to acute diarrhoea (20.0%). Fagundes-Neto and colleagues in Brazil reported a similar finding (33.3% and 18.2% in persistent and acute diarrhoea respectively) [5]. This supports the observation that the lactase enzyme is localized to the tips of the intestinal villi, a factor of clinical importance when considering the effect of diarrhoeal illness on the ability to tolerate lactose. Persistent diarrhoea also results in a more prolonged and extensive damage of the intestinal mucosa and the immature epithelial cells that replace these are often lactase deficient, leading to secondary lactase deficiency and lactose malabsorption [11]. Conversely, lactose intolerance prolongs and increases the severity of diarrhoea [15].

Thirty five children (70%) with lactose intolerance presented with perianal skin erosion (p < 0.001), a finding that has been documented [15]. In the presence of lactose intolerance, unabsorbed lactose gets metabolized by the colonic bacterial flora to organic acids and this is responsible for perianal skin erosion in children with diarrhoea [16].

While other studies have reported significant association of lactose intolerance with giardiasis [18,19], only one child in the current study was found to have giardiasis and she had no evidence of lactose intolerance. Possibly, this was because of a less sensitive method used (wet stool preparation), as the 'string test' of duodenal contents would have been more preferable. Six of the study participants had Cryptosporidia on modified ZN stain, only one of whom had evidence of lactose intolerance and this was not statistically significant. Similarly, in one patient an enteropathogen (salmonella non-typhi) was detected in stool cultures, with no evidence of lactose intolerance. None but one of the HIV positive patients in the study had been started on antiretroviral treatment during the study and she had no evidence of lactose intolerance.

Our study had limitations in that we did not use the breath hydrogen test which is the gold standard because it is expensive, cumbersome to use on a large scale and requires the patient to fast, in addition to use of a lactose load (procedures not desirable in severely malnourished children on highly regulated dietary management). It was not possible to exclude pre-existing/primary disturbances of lactose digestion, including chronic environmental entropathies. Other associated food allergies could not also be excluded. It was also not possible to determine a causal relationship between the different factors and lactose intolerance as this required a different study design.

## Conclusions

The prevalence of lactose intolerance in severely malnourished children with diarrhoea in the study setting of 25.5% is relatively high, especially in the 3-12 months age group. Clinical predictors of lactose intolerance in severely malnourished children included oedematous malnutrition, perianal skin erosion, higher mean stool frequency and having ≥2 diarrhoea episodes in the previous 3 months. Lactose intolerance should be considered and routine screening by stool pH and reducing substance undertaken in these children.

Use of lactose-free diets such as yoghurt should be considered for children found to have evidence of lactose intolerance and whose response on the standard therapy is poor.

## Abbreviations

MNU: Mwanamugimu Nutrition Unit; PIDC: Paediatric Infectious Disease Clinic; HIV: Human Immunodeficiency Virus; RCT: Routine Counseling and Testing; WHO: World Health Organization; ZN: Ziehl-Neelsen

## Conflict of interests

The authors declare that they have no competing interests.

## Authors' contributions

RN was the initiator of the study and contributed to the study design, data collection, and interpretation of results. IK, EM and HB contributed to the study design, interpretation of results and drafting of the manuscript. All authors have read and approved the final manuscript.

## Pre-publication history

The pre-publication history for this paper can be accessed here:

http://www.biomedcentral.com/1471-2431/10/31/prepub
